# Sub-national longitudinal and geospatial analysis of COVID-19 tweets

**DOI:** 10.1371/journal.pone.0241330

**Published:** 2020-10-28

**Authors:** Raphael E. Cuomo, Vidya Purushothaman, Jiawei Li, Mingxiang Cai, Timothy K. Mackey

**Affiliations:** 1 Department of Anesthesiology, San Diego School of Medicine, University of California, San Diego, California, United States of America; 2 Global Health Policy Institute, San Diego, California, United States of America; 3 Masters Program in Public Health, Department of Family Medicine and Public Health, University of California, San Diego, California, United States of America; 4 Department of Healthcare Research and Policy, University of California, San Diego, California, United States of America; Walter Sisulu University, SOUTH AFRICA

## Abstract

**Objectives:**

According to current reporting, the number of active coronavirus disease 2019 (COVID-19) infections is not evenly distributed, both spatially and temporally. Reported COVID-19 infections may not have properly conveyed the full extent of attention to the pandemic. Furthermore, infection metrics are unlikely to illustrate the full scope of negative consequences of the pandemic and its associated risk to communities.

**Methods:**

In an effort to better understand the impacts of COVID-19, we concurrently assessed the geospatial and longitudinal distributions of Twitter messages about COVID-19 which were posted between March 3rd and April 13th and compared these results with the number of confirmed cases reported for sub-national levels of the United States. Geospatial hot spot analysis was also conducted to detect geographic areas that might be at elevated risk of spread based on both volume of tweets and number of reported cases.

**Results:**

Statistically significant aberrations of high numbers of tweets were detected in approximately one-third of US states, most of which had relatively high proportions of rural inhabitants. Geospatial trends toward becoming hotspots for tweets related to COVID-19 were observed for specific rural states in the United States.

**Discussion:**

Population-adjusted results indicate that rural areas in the U.S. may not have engaged with the COVID-19 topic until later stages of an outbreak. Future studies should explore how this dynamic can inform future outbreak communication and health promotion.

## Introduction

Between the beginning of March 2020 and the end of April 2020, the number of recorded coronavirus disease 2019 (COVID-19) cases grew from under 100,000 to over 3 million worldwide [1]. Globally, growth has not been consistent, with recorded daily cases exhibiting noticeable but relatively muted spread in January, followed by low transmission in February, rapid daily increases in March, and high but sustained levels of new cases in April [2]. Similarly, the spread of COVID-19 has not had even geospatial distribution, with noticeably high levels of recorded cases in China, Western Europe, and New York; and relatively low levels of recorded cases in Sub-Saharan Africa and Central America [1], though the exact global burden of COVID-19 remains unknown.

Current evidence suggests that COVID-19 has an appreciable case-fatality rate when compared to other diseases of similar epidemiological characteristics and infectiousness [3]. However, establishing even basic epidemiological data during a health emergency can be challenging, particularly in the context of lack of sufficient access to testing and diagnostic capacity, overburdened health systems, and variability in clinical protocols for testing, contact tracing, and other public health measures [4]. A commonly-employed strategy to reduce deaths from COVID-19 when faced with unevenness in disease reporting is to prevent especially susceptible individuals, such as the elderly and immunocompromised [5], from contracting the virus by implementing social distancing guidelines [6]. Despite the rapid increase in number of deaths since the beginning of the COVID-19 pandemic, there has existed wide variability in the seriousness with which governments and local communities have pursued, implemented and enforced social distancing guidelines [7]. Concordantly, there may exist appreciable variability in the level of attention that local communities may have devoted to COVID-19 and associated prevention measures.

In addition, the COVID-19 pandemic has numerous ramifications which extend beyond physical health, including those which are sociocultural, economic, and psychological [8]. Individuals with certain mental health conditions or occupations may be adversely impacted by social distancing measures, though they may never contract COVID-19. Conversely, some individuals who become infected with COVID-19 may be entirely asymptomatic [9, 10]. Therefore, one additional reported case may not indicate any negative utility (if that person were asymptomatic), and case counts may not reflect all negative impacts of COVID-19 (including psychological and economic impacts). This lack of comprehensive COVID-19 burden is likely difficult to estimate, hence, necessitating alternative methodological approaches to assess interactions between outbreak trends from both a longitudinal and localized context.

The use of non-traditional forms of epidemiological data, particularly those that originate from online interaction by users, can provide additional insights how much communities pay attention to emerging public health issues, particularly when such data has resolution to geospatial location data and longitudinal data [11]. In this study we leverage data from the popular microblogging social media platform Twitter to rigorously describe the longitudinal and spatial distributions of COVID-19 social media engagement at sub-national levels for the United States. Analysis focused on longitudinal and geospatial assessments, both independently and concurrently, to better characterize community-level attention to the COVID-19 pandemic and related opportunities to engage the public in outbreak communication.

## Materials and methods

### Data collection

Tweets related to COVID-19 were collected prospectively from March 3rd to April 13th by using the Twitter public streaming Application Programing Interface (API) using a combination of cloud computing using virtual instances in Amazon Web Services (AWS) executed with scripts in the python programming language. Tweets were included if they contained three pieces of information: (1) geo-identifiable characteristics (e.g. geocoded metadata); and (2) keywords related to COVID-19, including: “corona outbreak,” “corona,” “anticorona,” “coronavirus,” “Wuhan virus,” “COVID,” “Wuhan pneumonia,” and “pneumonia of unknown cause.” Tweets were excluded if latitude and longitude coordinates were outside of land masses or if keywords were found outside the body of the tweet text.

Confirmed COVID-19 cases, deaths, and recoveries were obtained from Johns Hopkins University Github CSSEGISandData/COVID-19 repository [2]. These data were obtained for the United States at the secondary administrative level (i.e. counties). Active COVID-19 cases were based on diagnosed and reported cases of deaths and recoveries; specifically, active cases were calculated by subtracting a given day’s deaths and recoveries from confirmed cases, following the conventional approach used to compute active COVID-19 cases [12].

For normalization, population estimates for secondary administrative levels for the U.S. nationally were obtained from the American Community Survey [13].

### Data analysis

In an effort to remove less relevant tweets, such as those relating to news media, machine learning was used to train a classifier to detect tweets exhibiting accounts of first-hand experience with COVID-19. Training data was taken from another study which used tweets that were derived using the same set of keywords as this study [14]. The classifier was computed using support vector machines (SVM), utilizing a linear kernel algorithm via the *e1071* package in R, applied to a document-term matrix computed from the training set. All longitudinal and geospatial analyses utilized tweets detected using this machine learning classifier.

The C3 method of the Early Aberration Reporting System (EARS) was used to identify statistically significant aberrations in daily change in tweets related to COVID-19 for each of the fifty states in the United States. The EARS method is derived from a separate technique, the cumulative sum control chart (CUSUM) for sequential analysis. Furthermore, EARS is a syndromic surveillance approach used by the Centers of Disease Control and Prevention (CDC) and other public health agencies to detect statistically significant aberrations in infectious diseases [15]. All EARS methods involve comparison with preceding records, and the C3 method is the most sensitive of the three EARS methods. Specifically, the first eleven days of Twitter data was needed to establish a baseline for statistical comparison, and the earliest date it was possible to identify aberrations was March 14th. Logistically, a dataframe was created in R for each state, with a factor of dates and a series of observed tweets per day, and each day following the baseline period was assessed for statistically significant longitudinal aberration via the *surveillance* package.

Geospatial analyses involved the use of ArcGIS to create choropleth maps with nine intervals to visualize the geospatial distribution of tweets and tweets per capita at the secondary administrative level for the United States. Individual tweets were available as latitude/longitude point coordinates from the Twitter API, and these point coordinates were aggregated within county spatial polygons in ArcMap. For visualization of tweets per capita, the total number of tweets was divided by county-level population. Equal intervals were selected as a blue-yellow-red gradient in the “graduated colors” feature, thereby illustrating a consistent gradient of magnitudes.

Concurrent longitudinal and geospatial analysis was conducted by computing a space-time cube with 1-day time step intervals for 2,500 square-kilometer areas in the United States. The space-time cube is a construct whereby a record is produced for every combination of time step and space. After the space-time cube was created using built-in tools within ArcMap, the Emerging Hot Spot Analysis feature was used to analyze the space-time cube to compute the Getis Ord Gi* statistic for each area at each time point, thereby identifying of *z*-scores for hot spot and cold spot trends for each 2,500 square-kilometer area in the United States. Specifically, this methodology utilizes the Mann Kendall Trend Test to compute *z*-scores for every square area, thereby relaying the degree to which the given area was becoming “hotter” or “colder.” These *z*-scores were also visualized as a blue-yellow-red gradient so as to relay the distribution of longitudinal trends for tweets in all areas of the United States.

The Emerging Hot Spot Analysis feature was also conducted on space-time cubes for active daily COVID-19 cases per capita within the study time period. *Z*-scores for both COVID-19 cases per capita were then averaged for areas within each of the fifty states in the United States and the District of Columbia, as were *z*-scores for tweets per capita in each state. Ranking of average *z*-score was then computed for cases and tweets, and the largest discrepancies between rankings were reported.

Data management, statistical analysis, and geospatial visualization was conducted using R version 3.6.0 and ArcGIS version 10.6.

## Results

From March 3rd through April 13th, 173,847,058 tweets related to COVID-19 were collected. 1,244,478 of these tweets had geo-identifiable information which could be converted to point coordinates, of which 609,794 were from the United States. Of these US tweets, machine learning classification selected 17,841 with evidence of first-hand experience with COVID-19.

In an analysis to detect aberrantly high levels of tweets within the in the 32-day period between March 14th and April 13th, inclusive, approximately two-thirds of statistically significant aberrations at the state level were detected in the seven-day period between March 28th and April 3rd, inclusive (Table 1). Nearly all states with aberrantly high numbers of posts during this time period had relatively high proportions of rural populations. Specifically, within this seven-day period, Arkansas had four consecutive days of aberrantly high tweet volumes (March 28th through March 31st), with Mississippi and Missouri both having two consecutive days of high tweet volumes (March 29th and March 30th). Sunday, March 29th was the only day to exhibit aberrantly high tweet volumes for four separate states.

**Table 1:** Dates for significant aberrant levels of social media posts, by US state, utilizing the EARS C3 method. Columns prior to March 19th and after April 7th are not shown, as no aberrant dates were detected during this timeframe.

State	Tweets per Day	Aberrant Dates																			
Alabama	147									27-Mar	28-Mar										
Alaska	31																				
Arizona	357																				
Arkansas	49										28-Mar	29-Mar	30-Mar	31-Mar							
California	2346																				
Colorado	196																				
Connecticut	140																				
Delaware	47																				
Florida	1101																				
Georgia	515												30-Mar								
Hawaii	69																				
Idaho	49											29-Mar			1-Apr	2-Apr					
Illinois	474																				
Indiana	226																				
Iowa	93																3-Apr	4-Apr			
Kansas	98																				
Kentucky	173																				
Louisiana	215																				
Maine	42																				
Maryland	336																				
Massachusetts	334																				
Michigan	314																				
Minnesota	177													31-Mar	1-Apr						
Mississippi	72											29-Mar	30-Mar								
Missouri	215											29-Mar	30-Mar								
Montana	24	19-Mar				23-Mar															
Nebraska	70																				7-Apr
Nevada	214																				
New Hampshire	49																				
New Jersey	351																				
New Mexico	72										28-Mar										
New York	1264																				7-Apr
North Carolina	368																				
North Dakota	15																				
Ohio	450														1-Apr	2-Apr	3-Apr	4-Apr			
Oklahoma	106							25-Mar													
Oregon	183																				
Pennsylvania	513																				
Rhode Island	47																3-Apr	4-Apr			
South Carolina	169																				
South Dakota	17																				
Tennessee	294																				
Texas	1495																				
Utah	106																				
Vermont	19																				
Virginia	399	19-Mar	20-Mar			23-Mar															
Washington	380																				
West Virginia	42															2-Apr					
Wisconsin	146																				
Wyoming	8																				

**Fig 1:**
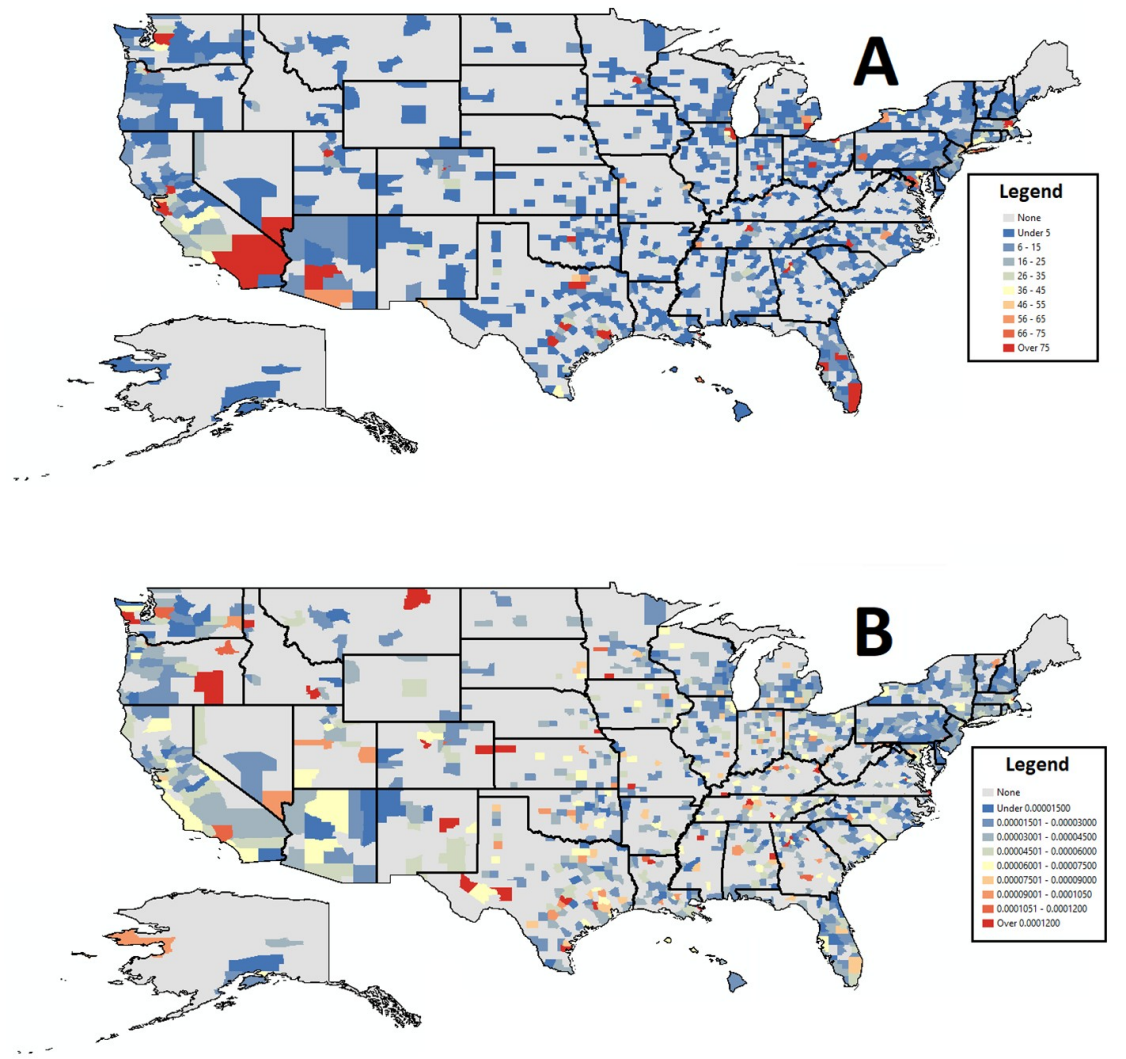
Between March 3rd and April 13th, US tweet counts (A) and US tweets per capita (B).

The number of tweets collected at the secondary administrative division in the United States were especially high in areas with high numbers of people (Fig 1A), with levels of tweets becoming much more evenly distributed after adjusting for population (Fig 1B).

**Fig 2:**
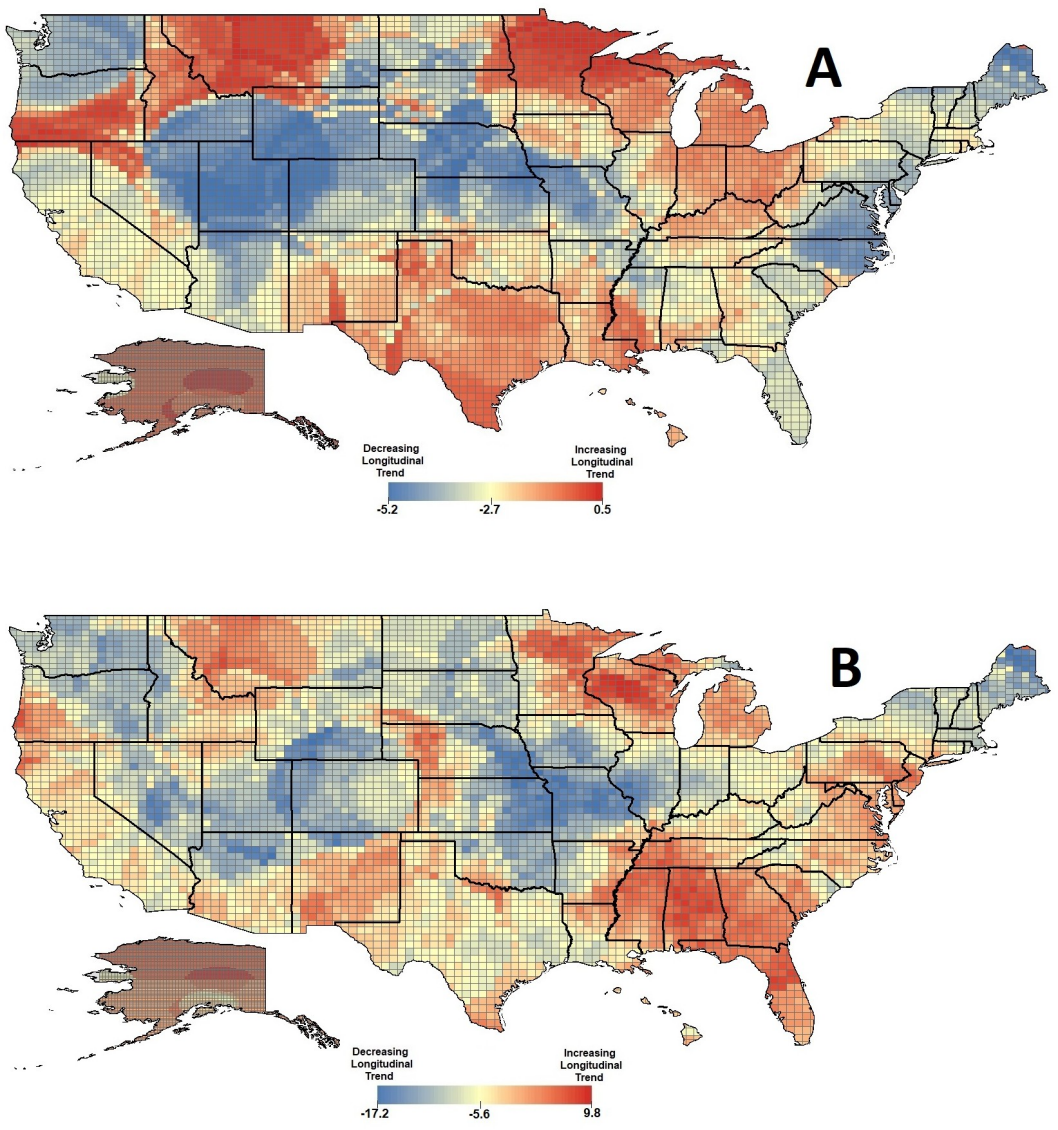
*Z*-scores for longitudinal trend in spatial hotpots of COVID-19 tweets (A) and COVID-19 tweets per capita (B) for the United States.

Similarly, time-space cubes were computed for the United States at 2,500 square-kilometer units with 1-day time step intervals, for both tweet counts over time and population-normalized tweets per capita over time. The number of tweets over time significantly decreased (*p* = 0.0008), and this trend remained consistent after normalizing for county-level population (*p* = 0.0012). However, a visualization of *z*-scores across US spatial units revealed discrepancies in trends toward significant hot/cold spots for (Fig 2) data, with appreciable discrepancies in the range of the distribution.

A time-space cube was computed for population-normalized US daily active cases of COVID-19, using county centroids with 1-day time step intervals. Overall, a statistically significant increase in county-level population-normalized COVID-19 cases was detected (*p* < 0.0001). *Z*-scores for trends from the resultant emerging hot spot analysis for COVID-19 cases per capita were compared to *z*-scores for trends from emerging hot spot analysis for COVID-19 tweets per capita. Average values were then computed for each state and the District of Columbia. The range of state-level *z*-scores observed for the cases per capita analysis was 1.12 (Alaska) to 8.85 (District of Columbia), and for the tweets per capita analysis was -10.78 (Missouri) to 1.89 (Alabama). Furthermore, ranks were also computed for trend *z*-scores of normalized cases and normalized tweets, with a rank of 1 for to the highest *z*-score value and a rank of 51 for the lowest *z*-score value. The largest discrepancies, in which a state had relatively high trend z-scores for increases in normalized cases while having relatively low trend z-scores for normalized tweets, were for New Hampshire (tweet rank of 43, case rank of 32); Vermont (tweet rank of 39, case rank of 8); and Rhode Island (tweet rank of 33, case rank of 4).

## Discussion

Our analyses uncovered a relative spike in tweets about COVID-19 around March 29th for predominantly rural areas within the United States. This spike in tweets immediately follows a period during which aberrantly higher number of cases of COVID-19 were reported in the United States. It may be that the rapid acceleration of reported cases caused increased concern about the pandemic, and that communities in these areas mostly ignored the public health threat until a relatively high threshold of cases were reported elsewhere. Alternatively, it is possible that the increased attention to COVID-19 in these communities was highly influenced by the social distancing guidelines and forced suspension of businesses that occurred during this same time period. This study suggests that, across subnational areas within the United States, there exists a highly variable threshold of perceived dangerousness and/or intrusiveness required to activate outbreak-related conversations on social media platforms such as Twitter, a finding that can inform future outbreak communication and health promotion strategies. Both of these causal scenarios may be greatly mediated by news media commonly more consumed in certain areas, and further study should be conducted to assess media-specific roles [11].

Concurrent geospatial and longitudinal analyses also indicate that predominantly rural areas of the United States increased engagement in COVID-19 social media conversations at later stages of the study timeframe. These analyses further suggest that there exists variability of engagement within states, as was observed for Idaho, Iowa, and South Dakota; and that regions of increasing concern can also span across multiple states, as was the case for the Montana-Idaho and for the Minnesota-Wisconsin areas. Interestingly, though several states exhibited a decline in social media engagement following relatively high points earlier in the study period, states eschewing this negative trend did not exhibit clear longitudinal patterns for recorded active COVID-19 cases. These results suggest that increased communication in certain areas may be a reaction to conditions unrelated to reported COVID-19 infection in their communities (such as discussion of social distancing guidelines or discussions of related government action). Alternatively, increased social media messaging about COVID-19 may result from higher numbers of people contracting COVID-19 than have been reported in these areas, which itself may be related to insufficient testing capacity and healthcare access, as has been widely reported and remains an acute problem in rural communities [16–18].

This study is unique in that it uses Twitter data as a proxy measure for assessing the concurrent longitudinal and geospatial distributions of attention to COVID-19 across local and regional communities in the United States. However, a number of studies have utilized similar methods in COVID-19 research, although usually for COVID-19 infection cases and/or deaths. For example, time-space methods were used to differentiate the trajectories of COVID-19 prevalence between rural and urban counties in the United States [19], and sequential spatial cross-sections have been assessed to define the evolution of hot spots in subnational regions [20]. While Twitter posts related to COVID-19 have been assessed using spatial and longitudinal techniques [21, 22], these studies have generally focused on country-level comparisons.

### Limitations

As these analyses were ecological in nature, this study is primarily intended to generate hypotheses, and the findings in this study should be further corroborated with other sources of data that can better identify specific COVID-19 hot and cold spots in relation to projected number of cases. Other data includes the availability and volume of COVID-19 testing kits, search engine query results (e.g. Google Trends data), COVID-19 hospitalizations data, etc. Tweets themselves were collected on the basis of containing certain keywords related to COVID-19, though the actual content of these tweets may vary in their interpretability for estimating attention to COVID-19 or disease burden. Future work by co-authors is focused on developing machine learning approaches to identify users reporting COVID-19-related symptoms, lack of testing access, and recovery, that could be used in a future study to better assess COVID-19 hot spots and also used to compare against aberrations in reporting.

### Conclusion

While preliminary, this study suggests that there may exist an appreciable degree of variability in the patterns defining community attention to public health topics, particularly as pertains to online discussion of infectious disease outbreaks. Further study should be conducted to characterize community-level moderators of these patterns and to understand the degree to which they may facilitate improved dissemination and impact of public health messaging. The use of EARS and time-space cubes appear to have utility in identifying longitudinal trends and their discrepancies across space, and further academic research on the topic of community-level attention to public health issues may seek to leverage these analytical techniques.

## Supporting information

S1 Data (CSV)

## References

[1] Dong E, Du H, Gardner L. An interactive web-based dashboard to track COVID-19 in real time. The Lancet Infectious Diseases. 2020. 10.1016/S1473-3099(20)30120-1 PMC7159018 32087114

[2] CSSE JHU. Novel coronavirus (covid-19) cases. GitHub Repository, https://github.com/CSSEGISandData/COVID-19, retrieved April. 2020;5:2020.

[3] Onder G, Rezza G, Brusaferro S. Case-fatality rate and characteristics of patients dying in relation to COVID-19 in Italy. Jama. 2020 10.1001/jama.2020.4683 32203977

[4] Buckee C. Improving epidemic surveillance and response: big data is dead, long live big data. The Lancet Digital Health. 2020;2(5):e218–e20. 10.1016/S2589-7500(20)30059-5 32518898 PMC7270775

[5] Cascella M, Rajnik M, Cuomo A, Dulebohn SC, Di Napoli R. Features, evaluation and treatment coronavirus (COVID-19). Statpearls [internet]: StatPearls Publishing; 2020. 32150360

[6] Lewnard JA, Lo NC. Scientific and ethical basis for social-distancing interventions against COVID-19. The Lancet Infectious diseases. 2020 10.1016/S1473-3099(20)30190-0 32213329 PMC7118670

[7] Andersen M. Early evidence on social distancing in response to COVID-19 in the United States. Available at SSRN 3569368. 2020.

[8] Sood S. Psychological effects of the Coronavirus disease-2019 pandemic. Research & Humanities in Medical Education. 2020;7:23–6.

[9] Tian S, Hu N, Lou J, Chen K, Kang X, Xiang Z, et al Characteristics of COVID-19 infection in Beijing. Journal of Infection. 2020. 10.1016/j.jinf.2020.02.018 PMC7102527 32112886

[10] Hu Z, Song C, Xu C, Jin G, Chen Y, Xu X, et al Clinical characteristics of 24 asymptomatic infections with COVID-19 screened among close contacts in Nanjing, China. Science China Life Sciences. 2020:1–6. 10.1007/s11427-020-1661-4 32146694 PMC7088568

[11] Eysenbach G. Infodemiology and infoveillance: framework for an emerging set of public health informatics methods to analyze search, communication and publication behavior on the Internet. Journal of medical Internet research. 2009;11(1):e11 10.2196/jmir.1157 19329408 PMC2762766

[12] Rai B, Shukla A, Dwivedi LK. COVID-19 in India: Predictions, Reproduction Number and Public Health Preparedness. medRxiv. 2020.

[13] U.S. Census Bureau. American Community Survey 5-Year Public Use Microdata Samples 2019. https://factfinder.census.gov/.

[14] Mackey TK, Purushothaman V, Li J, Shah NS, Nali M, Bardier C, et al Machine Learning to Detect Self-Reporting of COVID-19 Symptoms, Testing Access and Recovery on Twitter. [Under Review]. 2020. 10.2196/19509 PMC7282475 32490846

[15] Fricker RD Jr, Hegler BL, Dunfee DA. Assessing the performance of the early aberration reporting system (EARS) Syndromic Surveillance Algorithms. Stat Med researchgate net. 2007.

[16] Brännström Å, Sjödin H, Rocklöv J. Predicting the number of COVID-19 cases from the reported number of deaths. 2020.

[17] Catalyst N. Lessons from CEOs: Health Care Leaders Nationwide Respond to the Covid-19 Crisis. NEJM Catalyst Innovations in Care Delivery. 2020;1(2).

[18] Keshava SN, Gupta A, Pant R, Sutphin PD, Kalva S. Practice of Interventional Radiology during the COVID-19 Pandemic. Journal of Clinical Interventional Radiology ISVIR. 2020.

[19] Paul R, Arif AA, Adeyemi O, Ghosh S, Han D. Progression of COVID-19 From Urban to Rural Areas in the United States: A Spatiotemporal Analysis of Prevalence Rates. The Journal of Rural Health. 2020 10.1111/jrh.12486 32602983 PMC7361905

[20] Ramírez IJ, Lee J. COVID-19 Emergence and Social and Health Determinants in Colorado: A Rapid Spatial Analysis. International Journal of Environmental Research and Public Health. 2020;17(11):3856 10.3390/ijerph17113856 32485854 PMC7312929

[21] Singh L, Bansal S, Bode L, Budak C, Chi G, Kawintiranon K, et al. A first look at COVID-19 information and misinformation sharing on Twitter. arXiv preprint arXiv:200313907. 2020.

[22] Chen N, Zhong Z, Pang J. An Exploratory Study of COVID-19 Information on Twitter in the Greater Region. arXiv preprint arXiv:200805900. 2020.

